# Sugar Beverages and Dietary Sodas Impact on Brain Health: A Mini Literature Review

**DOI:** 10.7759/cureus.2756

**Published:** 2018-06-07

**Authors:** Ibrar Anjum, Syeda S Jaffery, Muniba Fayyaz, Abdullah Wajid, Armghan H Ans

**Affiliations:** 1 Internal Medicine, The University of Texas MD Anderson Cancer Center, Houston, USA; 2 Department of Research, California Institute of Behavioral Neurosciences & Psychology, Skokie, USA; 3 Department of Internal Medicine, Fatima Memorial Hospital, Lahore, PAK; 4 FMH College of Medicine & Dentistry, Lahore, PAK; 5 Department of Cardiology, University of Pennsylvania, Philadelphia, USA

**Keywords:** soda and other drinks role in sleep, effects of soda and dietary consumptions on brain, soft drinks effect on congnitive ability, caffeine and brain, sugar-sweetened beverages and its effect on mind

## Abstract

Sugar-sweetened beverages containing caffeine are widely used among humans nowadays and can have negative consequences on the overall health. Our study aims to discuss the effects of these sugar-sweetened beverages (SSB) and how they can impact the health in different ways particularly on the brain. Some of the mechanisms by which soft drinks can exert adverse effects include an increase in glutathione-6-dehydrogenase level, increased levels of gamma-aminobutyric acid (GABA), glutamate and dopamine alteration in brain waves on electroencephalography (EEG) eventually leading to stroke and dementia. They can increase the oxidative stress by a decreasing monoamine oxidase and acetylcholine esterase and antioxidants such as glutathione and catalase. The sleep quality and duration of sleep is also significantly affected by their increased consumption. Also, the consumption of sodium benzoate (found in beverages) on impairing memory, motor coordination, affecting reduced glutathione (GSH), increasing the malondialdehyde (MDA) level in the brain and producing attention deficit hyperactivity disorder (ADHD) in children is emphasized. Finally, we will highlight how diet drinks can also be harmful and the maternal consumption of chocolate or soft drinks during pregnancy and postnatal period can be linked to cognitive impairment and child obesity.

## Introduction and background

In today's advancing society, the use of soda beverages has become necessary if not somewhat unavoidable. Whether the beverage consists of carbonated-caffeine drinks such as cola or diet drinks like diet cola, everyone enjoys the pleasurable and refreshing taste of a fizzy drink. While these fizzy drinks may have their uplifting and thirst-quenching satisfaction, they correspondingly play a role in hampering and impacting the normal physiological functioning of the brain [1]. The main component in carbonated-soft drinks is caffeine which triggers the excitation of the reticular system within the brain. Excess excitation of the system leads to insomnia, psychomotor agitation and headaches [2]. These drinks not only provide a refreshing and pleasurable taste but they also come with significant liability. El‑Terras et al. conducted a study on Wistar rats for evaluating chronic soda use to gene expression. The analysis reported significant imbalance and altered gene expression also known as oxidative stress within the brain including decreased levels of monoamine oxidase and acetylcholine esterase enzymes. These rats also exhibited decreased levels of antioxidants including Catalase and Glutathione Reductase [3]. Though many prefer diet drinks to regular carbonated sodas for their decreased health risks, in a study published by European Journal of Clinical Nutrition, aspartame, the main ingredient used in diet drinks, was linked to insomnia, headaches, and seizures and in chronic cases blindness, neurotoxicity and memory loss [4,5]. The reason behind is the breakdown of aspartame into phenylalanine, aspartic acid, and methanol, the main components of aspartame. These breakdown products although play a vital role in the body's growth and development. The excess accumulation of these components due to diet drink intake results in cytotoxic effects [6]. Whether the use of carbonated-caffeine cola or diet cola, artificially flavoured drinks and their increased caloric intake demonstrate and support brain dysfunction, dementia and stroke [7].

## Review

Soda and dietary consumption in today’s era

Sugar-sweetened beverages (SSBs) are the leading sources of added sugars in the diet and are increasing on a global level. Caffeine is the central part of a human’s diet and is also one of the main components of many foods and beverages, including coffee, tea, soft drinks, dietary drinks, energy drinks and its related products. According to World Health Organization (WHO) Guideline 2015 on the intake of free sugars, a single can of sugar-sweetened soda contains about the upper limit of the recommended 25–50 grams per day [8].

Effect of caffeine on brain

Caffeine is a stimulant frequently consumed by adults and children. Caffeine consumption between 100 and 400 mg has been associated with an increased risk of nervousness, jitteriness, and fidgetiness. Because of continued brain development involving myelination and pruning processes in children, it is harmful to their health. Owolabi et al. conducted a study on rats by administering caffeine and cannabis. It resulted in increased level in glutathione-6-phosphatase dehydrogenase. Also, glutamate, gamma-aminobutyric acid (GABA), and dopamine were increased [9]. A recent study conducted by Xu and Reichelt on rats observed the effects of caffeinated beverages and sucrose on the brain. The study concluded that both ingredients affect the brain causing anxiety-like changes and altering the level of parvalbumin (a calcium-binding protein) in the hippocampus which acts as the second messenger, organizing microtubules. Its alteration in the brain is noticed in patients with Alzheimer’s disease [10]. Pase et al. conducted a prospective cohort study on consumption of artificially sweetened drinks. The study revealed that artificially sweetened drinks are associated with a higher risk of stroke and dementia [11]. Free sugars are also the essential ingredient in drinks and essential cause of dental caries [12]. These include monosaccharides like glucose, fructose and disaccharides like sucrose or table sugar which are added to foods and drinks by the manufacturer, cook or consumer [13]. In a recent survey, 48% underestimated the content of fruit juices and smoothies on average, whereas the sugar content of carbonated drinks was overestimated by 12% [14]. Also, Meng et al. conducted a study which showed the effect of drinks like coke and other soft drinks on the brain. They investigated the effects of soft drinks and regular coffee on electroencephalography (EEG) signals under resting state and on the performance of motor imaginary-based brain-computer interface. It was concluded that these drinks affect brain waves in different patterns [15]. Pase et al. contributed new data to this debate using prospective data from the Framingham Offspring Cohort study. They investigated the relationship between recent and long-term consumption of sugar, artificially sweetened beverages and the risks of incident stroke and dementia [16]. Furthermore, a recent study by Lebda et al. was done on rats to understand the effect of aspartame in soft drink on brain function and its association between energy status in the brain, oxidative stress and molecular pathways of apoptosis. It was concluded that it affects rat’s brain energy function by inhibiting serum and brain creatinine kinase. It also alters electrolytes by increasing calcium and sodium and decreasing copper, iron, zinc, and potassium. Furthermore, soft drinks can also affect hormonal levels in the blood by increasing thyroid hormone T4 and parathyroid hormones and lowering thyroid hormone T3 and aldosterone levels [17].

Role of drinks in sleep patterns

Consumption of soft drinks and other beverages is mostly seen in children and young adults. It affects sleep pattern and its quality especially in students [18]. A study conducted by Marmorstein investigated how caffeinated drinks affect sleep in young adolescents. He found that young adolescents who consume energy drinks and coffee are at unusually high risk of alcohol abuse [19]. Also, a recent study conducted by Chaput et al. illustrated that short sleep duration among children is associated with a longer intake of regular soft drink and earlier bedtimes are associated with regular use of soft drinks and other beverages [20].

Drinks consumption and its effect on children

Nowadays, children are getting 30% of sugar intake by soft drinks on a regular basis [21]. Sodium benzoate (SB) is found in many food and beverages. Its increased consumption especially in soft drinks and other beverages in children links to attention deficit-hyperactivity disorder. A study conducted by Khoshnoud et al. investigates the effect of different levels of SB on mice brain. It was concluded that SB significantly impairs memory and motor coordination, reduces glutathione (GSH) level and increases the malondialdehyde (MDA) level in the brain. However, some alteration was observed in the Acetylcholinesterase (AChE) Activity. These findings suggest that short-term consumption of SB can impair memory performance and increase brain oxidative stress in mice [22].

Use of soft and dietary drinks during pregnancy and its impact on offspring and children

A study was conducted by Kjaergaard et al. in which he concluded the effect of maternal intake of chocolate and soft drink and its long-term consequences for the metabolic phenotype in the offspring if they continue to take high sucrose soft drink supplement diet in postnatal life. These offspring showed signs of obesity despite lowered energy intake associated with alterations in the hypothalamic leptin signalling [23]. Also, a study conducted by Cohen et al. shows sugar consumption, especially from SSBs during pregnancy and childhood, may adversely affect child cognition. However, healthy food and nutrition intake can prevent it and may lead to improvement of cognition [24].

## Conclusions

In conclusion, the overall consumption of soft drinks containing sugar and caffeine is increasing drastically worldwide, especially among the young adults. Whether it is regular or diet soda, they all have been known to produce mechanisms harming the human body. Therefore, this issue should be emphasized widely, and more awareness should be raised to decrease its further use to improve the quality of life thus eliminating its negative consequences. Overall, more research is still required regarding this topic.
